# Functional expression of two system A glutamine transporter isoforms in rat auditory brainstem neurons

**DOI:** 10.1016/j.neuroscience.2009.09.015

**Published:** 2009-12-15

**Authors:** A. Blot, D. Billups, M. Bjørkmo, A.Z. Quazi, N.M. Uwechue, F.A. Chaudhry, B. Billups

**Affiliations:** a Department of Pharmacology, University of Cambridge, Tennis Court Road, Cambridge CB2 1PD, UK; b The Biotechnology Centre of Oslo, University of Oslo, Blindern, N-0317 Oslo, Norway; c The Centre for Molecular Biology and Neuroscience, University of Oslo, Blindern, N-0317 Oslo, Norway

**Keywords:** Slc38a1, Slc38a2, SAT1, SAT2, SNAT1, SNAT2, APV, dl-2-amino-5-phosphonopentanoic acid, BCH, 2-aminobicyclo-[2.2.1]-heptane-2-carboxylic acid, dl-TBOA, dl-threo-b-benzyloxyaspartate, EAAT, excitatory amino acid transporter, GST, glutathione-S-transferase, I/V, current–voltage, I_gln_, glutamine-induced current, I_pro_, proline-induced current, MeAIB, N-(methylamino)isobutyric acid, MK801, dizocilpine maleate, MNTB, medial nucleus of the trapezoid body, NBQX, 2,3-Dioxo-6-nitro-1,2,3,4-tetrahydrobenzo[f]quinoxaline-7-sulfonamide, SAT1, system A transporter 1, SAT2, system A transporter 2, TCA, tricarboxylic acid, TTX, tetrodotoxin, VGLUT, vesicular glutamate transporter

## Abstract

Glutamine plays multiple roles in the CNS, including metabolic functions and production of the neurotransmitters glutamate and GABA. It has been proposed to be taken up into neurons via a variety of membrane transport systems, including system A, which is a sodium-dependent electrogenic amino acid transporter system. In this study, we investigate glutamine transport by application of amino acids to individual principal neurons of the medial nucleus of the trapezoid body (MNTB) in acutely isolated rat brain slices. A glutamine transport current was studied in patch-clamped neurons, which had the electrical and pharmacological properties of system A: it was sodium-dependent, had a non-reversing current-voltage relationship, was activated by proline, occluded by N-(methylamino)isobutyric acid (MeAIB), and was unaffected by 2-aminobicyclo-[2.2.1]-heptane-2-carboxylic acid (BCH). Additionally, we examined the expression of different system A transporter isoforms using immunocytochemical staining with antibodies raised against system A transporter 1 and 2 (SAT1 and SAT2). Our results indicate that both isoforms are expressed in MNTB principal neurons, and demonstrate that functional system A transporters are present in the plasma membrane of neurons. Since system A transport is highly regulated by a number of cellular signaling mechanisms and glutamine then goes on to activate other pathways, the study of these transporters *in situ* gives an indication of the mechanisms of neuronal glutamine supply as well as points of regulation of neurotransmitter production, cellular signaling and metabolism in the native neuronal environment.

Glutamine is the most abundant amino acid in the interstitial fluid of the CNS, with a concentration in the range of 0.2–0.5 mM (Jacobson et al., 1985; Reichel et al., 1995; Kanamori and Ross, 2004), which is over an order of magnitude higher than any other amino acid. It is not known to have any direct neurotransmitter action, but does play several important roles within the CNS including production of proteins, pyrimidine nucleotides, excitatory and inhibitory neurotransmitters and tricarboxylic acid (TCA) cycle intermediates. The intracellular glutamine concentration is thought to be in the millimolar range, and transport into neurons occurs against a substantial concentration gradient, which requires energy-dependent transport proteins (Erecinska and Silver, 1990).

The main transport systems for glutamine are systems A, ASC, B^0^, B^0,+^, b^0,+^, L, N, and y^+^L (reviewed by Bode, 2001; Broer, 2008). Systems ASC, b^0,+^, L and y^+^L are obligate exchangers, swapping internal and external amino acids across the plasma membrane. In contrast, systems A, B^0^, and B^0,+^ use the energy derived by the sodium gradient to power the uptake of amino acids in an electrogenic manner, without counter-transporting another amino acid. System N co-transports sodium and counter-transports a proton, resulting in a transport process that is close to energetic equilibrium and can mediate the uptake or release of amino acids under physiological conditions (Chaudhry et al., 1999; Broer et al., 2002). Glutamine transporter expression in the CNS has been observed for system A isoforms SAT1 (also called SA2, SNAT1, NAT2, GlnT and ATA1: Varoqui et al., 2000; Wang et al., 2000; Chaudhry et al., 2002b; Melone et al., 2004) and SAT2 (also called SA1, SNAT2 and ATA2: Reimer et al., 2000; Sugawara et al., 2000a; Yao et al., 2000; Gonzalez-Gonzalez et al., 2005; Melone et al., 2006; Jenstad et al., 2009); system ASC (ASCT2: Broer et al., 1999; Dolinska et al., 2004; Yamamoto et al., 2004); system B^0^ (B0AT2: Inoue et al., 1996; Takanaga et al., 2005; Broer et al., 2006); system B^0,+^ (ATB^0,+^: Sloan and Mager, 1999); system b^0,+^ (rBAT/b^0,+^AT: Bertran et al., 1992; Tate et al., 1992; Wells and Hediger, 1992); system L (LAT1 and LAT2: Kanai et al., 1998; Rossier et al., 1999; Segawa et al., 1999); system N (SN1/SNAT3 and SN2/SNAT5: Chaudhry et al., 1999, 2001; Nakanishi et al., 2001; Boulland et al., 2002, 2003; Cubelos et al., 2005) and system y^+^L (y^+^LAT2: Broer et al., 2000).

As most studies use radiolabelled substrates to study the properties of transport systems in bulk tissue or cultured cells, the cellular localization and functional properties of these different transporters *in vivo* is uncertain. To investigate glutamine transport in individual, identified neurons in their native physiological environment, we recorded amino acid-induced transporter currents using whole-cell patch-clamping in acutely isolated rat brain slices. *In situ* recordings were made from principal neurons in the medial nucleus of the trapezoid body (MNTB), which are neurons in the auditory brainstem that receive mainly excitatory glutamatergic input, and release glycine, GABA and glutamate at synapses in the adjacent medial and lateral superior olives (MSO and LSO; Spangler et al., 1985; Adams and Mugnaini, 1990; Bledsoe et al., 1990; Chaudhry et al., 1998; Gillespie et al., 2005). These cells have a spherical cell body with only a small dendritic tree (Smith et al., 1998; Leao et al., 2008), which allows for precise recording of somatic currents and eliminates artifacts due to dendritic filtering (Williams and Mitchell, 2008). The astrocytes surrounding the principal neurons in the MNTB have been shown to strongly express the system N transporters SN1 and SN2 (Boulland et al., 2002; Cubelos et al., 2005), which are thought to be responsible for the export of glutamine from the glial compartment (Chaudhry et al., 1999). System N and system A transporters in adjacent cells have been proposed to form a system N–system A shuttle (Chaudhry et al., 2002a; Gammelsaeter et al., 2009; Jenstad et al., 2009), which would transfer glutamine from glia to neurons. In support of this hypothesis, our electrophysiological and immunocytological data show that MNTB principal neurons express functional system A glutamine transporters on their soma. This provides a valuable insight into the possible mechanisms that these neurons employ for amino acid and neurotransmitter metabolism.

## Experimental procedures

### Slice preparation

Brain slices were obtained from 10 to 15 day old Wistar rats, killed by decapitation in accordance with the UK Animals (Scientific Procedures) Act 1986. All animal experiments were approved by the relevant local authorities (University of Cambridge, UK and University of Oslo, Norway), and every effort was taken to reduce the number of animals used and to minimize any suffering. Brains were quickly removed into a solution at approximately 0 °C containing (in mM) 250 sucrose, 2.5 KCl, 10 glucose, 1.25 NaH_2_PO_4_, 26 NaHCO_3_, 4 MgCl_2_, 0.1 CaCl_2_; gassed with 95% O_2_ +5% CO_2_ (pH 7.4). Transverse brainstem slices, approximately 150 μm thick, were cut using an Integraslice 7550PSDS (Campden Instruments, Loughborough, UK), and slices were placed in an incubation chamber maintained at 37 °C for half an hour, before being allowed to cool to room temperature and used within the next 6 h. The incubation chamber contained artificial cerebrospinal fluid (aCSF), which comprised (in mM) 125 NaCl, 2.5 KCl, 10 glucose, 1.25 NaH_2_PO_4_, 26 NaHCO_3_, 1 MgCl_2_, 2 CaCl_2_; gassed with 95% O_2_ +5% CO_2_ (pH 7.4).

### Electrophysiological recording

MNTB neurons were visualized with infrared differential interference contrast (IR–DIC) optics on a Nikon E600FN microscope (Nikon Corporation, Tokyo, Japan) with a 60×, numerical aperture 1.0, water immersion, fluor lens. Slices were perfused at a rate of approximately 1 ml/min with aCSF (as above) at a temperature of 31–35 °C. In all experiments, except for Fig. 4A–C, a cocktail of channel inhibitors was added to the recording solution, containing (in μM) 40 dl-2-amino-5-phosphonopentanoic acid (APV), 10 dizocilpine maleate (MK801), 10 bicuculline, 1 strychnine, 1 TTX (tetrodotoxin) and 20 2,3-dioxo-6-nitro-1,2,3,4-tetrahydrobenzo[f]quinoxaline −7-sulfonamide (NBQX). In the experiments recorded in 0 mM sodium solution (Fig. 3), NaCl and NaHCO_3_ were replaced by choline chloride and choline bicarbonate, respectively, and NaH_2_PO_4_ was replaced by KH_2_PO_4_ (with a corresponding reduction in KCl concentration and increase in choline chloride concentration to balance the other ions). Whole-cell patch-clamp recordings were made from MNTB cells using thick-walled glass pipettes (GC150F-7.5; Harvard Apparatus, Edenbridge, Kent, UK) with a HEKA EPC-10 double amplifier and Patchmaster software (HEKA Elektronik Dr. Schulze GmbH; Lambrecht/Pfalz, Germany). The intracellular solution contained (in mM) 110 Cs-methanesulfonate, 40 4-(2-hydroxyethyl) piperazine-1-ethanesulfonic acid (HEPES), 10 tetraethylammonium chloride (TEA), 5 Na_2_-phosphocreatine, 20 sucrose, 0.2 ethylene glycol-bis(2-aminoethylether)-N,N,N′,N′-tetraacetic acid (EGTA), 2 MgATP, 0.5 NaGTP, and 8 μM CaCl_2_ (pH 7.2 with ∼10 mM CsOH). A measured liquid junction potential of 3 mV was not corrected for. Recording pipette open-tip resistances were 5–8 MΩ and whole-cell access resistances were <30 MΩ. Lucifer Yellow (<0.05%) was added to the internal solution to allow confirmation of the post synaptic recording site by fluorescent imaging following the completion of the experiment. MNTB cells were voltage-clamped at −70 mV (unless stated) and currents were filtered at 10 and 2.9 kHz, and digitized at 25 kHz. Glutamine (or other amino acids) were dissolved in the relevant external solution and applied by pressure ejection (5–10 p.s.i.) from a patch pipette (as above, open tip resistance 4–6 MΩ) using a Picospritzer II (General Valve, Fairfield, NJ, USA). Puff applications were repeated at 30 s intervals. When applying two different glutamine concentrations, or glutamine in different external solutions, two puffer pipettes were used. The pipettes were a pair pulled from one piece of glass and therefore had the same tip diameter, were attached to the same pressure source and were placed equidistant from the cell.

### Immunostaining

Male Wistar rats, 200–300 g, were anesthetized with pentobarbital and subjected to transcardial perfusion fixation with 4% paraformaldehyde in 0.1 M sodium phosphate buffer pH 7.4. The brains were dissected out and kept in fixative over night. 40–50 μm thick coronal sections were cut by a vibratory microtome. Immunoperoxidase staining was done as described previously (Chaudhry et al., 1998; Boulland et al., 2004). Briefly, free-floating sections were treated with 1 M ethanolamine (pH 7.4), followed by incubation with 1% H_2_O_2_ in phosphate-buffered saline (PBS). The sections were incubated in solution of 10% fetal bovine serum and 0.1% NaN_3_ in buffer A (0.3 M NaCl, 0.1 M Tris-HCl pH 7.4, 0.05% Triton X-100) prior to incubation with the primary antibodies in the same solution. Following incubation in biotinylated secondary antibodies and the streptavidin-biotinylated horseradish peroxidase complex, the immunoreaction was demonstrated by addition of 3,3′-diaminobenzidine (0.5 mg/ml) in PBS activated by H_2_O_2_.

The affinity-purified antibodies against SAT1 and SAT2 have been characterized and were used as described previously (Buntup et al., 2008; Jenstad et al., 2009). SAT1 was used at a final concentration of 1 μg/ml, while SAT2 was used at a concentration of 10 μg/ml. The specificity of the immunoreaction was confirmed by pre-incubating the antibodies with the corresponding glutathione-S-transferase (GST) fusion protein (30–100 μg) used for immunization of the rabbits.

For fluorescent immunocytochemistry, 13–14 day old rats were used and stained as previously described (Billups, 2005). SAT1 and SAT2 antibodies (as above) were used at 0.5 and 0.25 μg/ml respectively, and secondary fluorescent antibodies (Alexa Fluor 488, 2 μg/ml; Invitrogen, Carlsbad, CA, USA) were visualized with a confocal microscope (Leica SP5; Leica Microsystems CMS GmbH, Mannheim, Germany). Tissue was mounted in Vectashield with DAPI (4′,6-diamidino-2-phenylindole; Vector Laboratories, Burlingame, CA, USA) to label nuclei for positive identification of MNTB cells.

### Data analysis

**Fig. 1:**
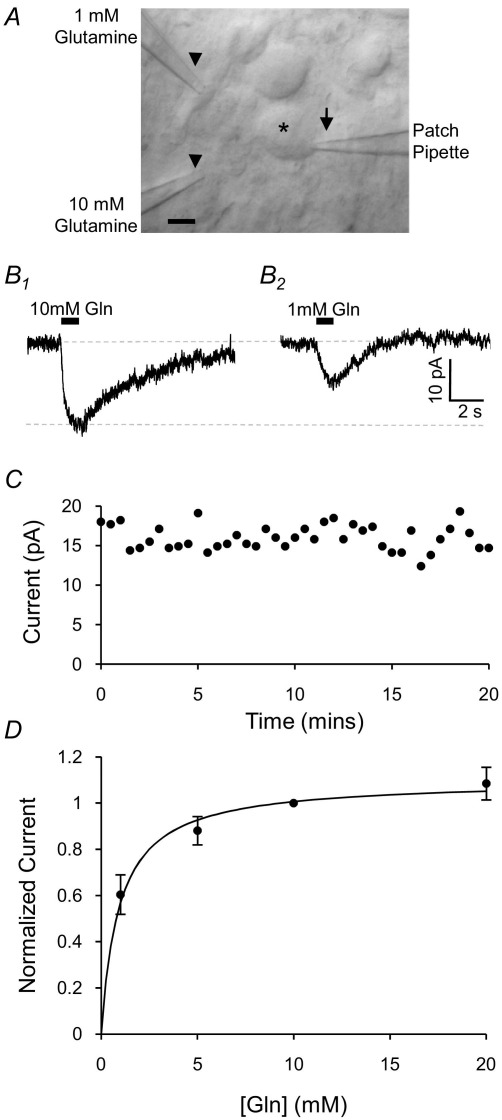
Glutamine-induced currents (I_gln_) are observed in whole-cell voltage-clamped MNTB neurons. (A) Differential interference contrast image of a patch-clamped MNTB neuron in the brain slice. The cell (*), patch pipette (arrow) and two puffer pipettes (arrow heads) are shown. The scale bar is 10 μm. (B): Puff-application of 10 mM glutamine for 1 s induces an inward membrane current of −20.6±0.9 pA (B_1_; *n*=61) in MNTB neurons whole-cell voltage-clamped at −70 mV. Application of 1 mM glutamine from the other puffer pipette induces a current 60±9% of the current induced by 10 mM glutamine (B_2_; *n*=3). (C) Plot of the magnitude of I_gln_ over time, demonstrating the temporal stability of the current. (D) Dose–response curve of I_gln_, normalized to I_gln_ at 10 mM glutamine. The data are best-fit with a Michaelis–Menten curve (*K*_m_=0.94±0.25 mM, V_max_=1.10±0.04, *R*^2^=0.60) shown by the solid line.

**Fig. 2:**
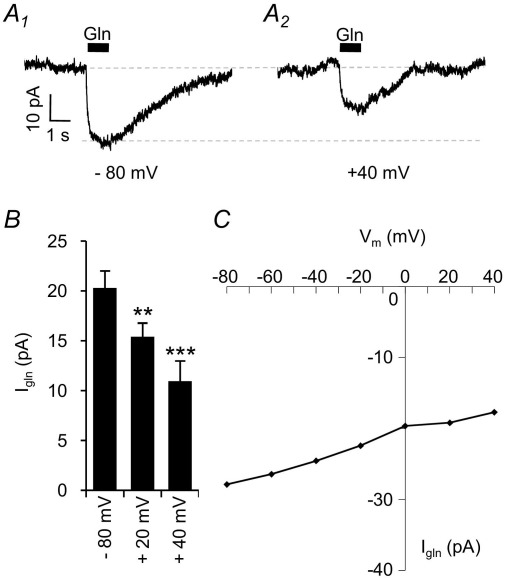
I/V relationship of I_gln_ indicates activation of a transporter. (A) Currents elicited by 10 mM glutamine application at holding potentials of −80 mV (A_1_) and +40 mV (A_2_). (B) I_gln_ at −80 mV (−20.3±1.7 pA; *n*=6) is reduced to −15.4±1.4 pA at +20 mV (*n*=6; *P*<0.01 indicated by the asterisks) and further reduced to −11.0±2.0 pA at +40 mV (*n*=6; *P*<0.001 indicated by the asterisks). (C) Sample current–voltage relation for 1 cell showing a reduced current at positive potentials, but no outward current.

**Fig. 3:**
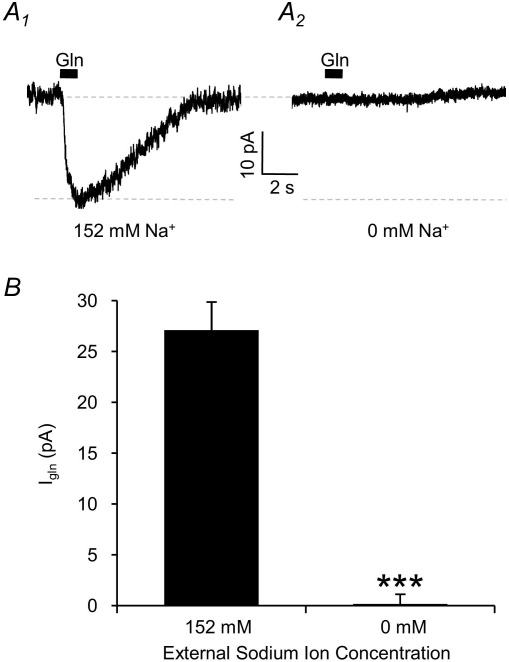
The glutamine transport current is eliminated by removing external sodium ions. (A) I_gln_ in control solution containing 152 mM Na^+^ (A_1_) is abolished by complete removal of Na^+^ from the external medium (A_2_). (B) Averaged data showing that I_gln_ is absolutely dependent on the presence of external sodium (*n*=7; *P*<0.001 indicated by the asterisks).

**Fig. 4:**
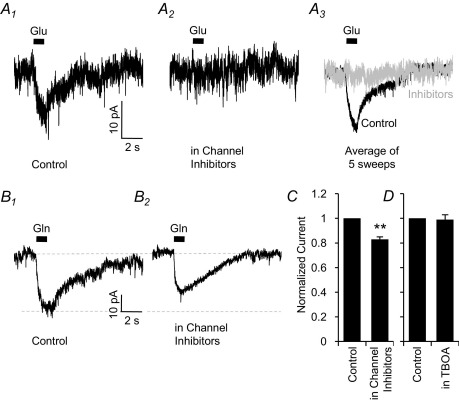
Glutamate receptor and transporter currents do not contribute to the observed glutamine current. (A) Puff application of 5 μM glutamate induces an inward current (A_1_; −7.6±0.9 pA; *n*=5), which is completely eliminated (A_2_; −0.1±0.1 pA; *n*=5; *P*<0.001) by bath application of a cocktail of ion channel inhibitors containing 40 μM APV, 10 μM MK801, 10 μM bicuculline, 1 μM strychnine, 1 μM TTX and 20 μM NBQX. (B): Without glutamate, GABA and glycine receptor inhibitors in the bathing solution, I_gln_ was −24±3 pA (C_1_; *n*=7). Bath application of the channel inhibitors reduces I_gln_ to −20±2 pA (C_2_; *n*=8; *P*<0.01). All other electrophysiological experiments (Figs. 1–5) were done in the presence of this cocktail of inhibitors. (C): The fractional reduction of I_gln_ by the channel inhibitors is 17±2% (*n*=8; *P*<0.01 shown by the asterisks), indicating only a minor contamination current. (D): Application of 200 μM dl-TBOA, to inhibit glutamate transporters, has no effect on I_gln_ recorded in the presence of the channel inhibitors. I_gln_ in control was −14.9±2.4 pA and in TBOA was −14.5±1.4 pA (99±4% of control; *n*=4, *P*>0.05).

**Fig. 5:**
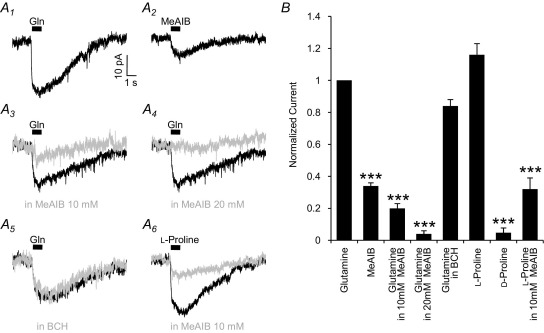
I_gln_ is mediated by system A transporters. (A) Compared to I_gln_ (A_1_), the transported system A analogue MeAIB (10 mM) induces a reduced current of −7.4±0.8 pA (A_2_; *n*=4; *P*<0.001). In the presence of 10 mM (A_3_, grey trace) or 20 mM MeAIB (A_4_, grey trace), I_gln_ (black traces) is inhibited. The presence of 10 mM BCH (A_5_, grey trace), which inhibits B^0^ transporters, does not reduce I_gln_ (A_5_, black trace). Puff-application of 10 mM l-proline (A_6_, black trace) produces a transport current of comparable magnitude to 10 mM glutamine, which is similarly inhibited by 10 mM MeAIB (grey trace). B: Normalized data showing the relative magnitude of the MeAIB induced current (34±2%; *n*=4; *P*<0.001), I_gln_ inhibition by 10 mM MeAIB (80±3% reduction; *n*=15; *P*<0.001) and 20 mM MeAIB (96±2%; *n*=4; *P*<0.001). Ten millimolar BCH had no significant effect on I_gln_ (16±4% reduction; *n*=6; *P*>0.05). The l-proline-induced current is a similar magnitude to I_gln_, (*n*=12; *P*>0.05), and it is inhibited by 72±6% by the addition of 10 mM MeAIB to the bath (*n*=4, *P*<0.001). d-proline induces a current that is much smaller (5±3%) than the l-proline current (*n*=5; *P*<0.001). *P* values of <0.001 are indicated by the asterisks over the appropriate bars.

Current traces were measured using Patchmaster. For small currents (<5 pA) at least three traces were averaged to improve the signal-to-noise ratio before measurement. Data are presented as mean±SEM and regarded as statistically significant if *P*<0.05 using a two-tailed paired Student's *t*-tests. For multiple comparisons (data from Figs. 3B, 4D, 5B combined), one-way analysis of variance (ANOVA) was used, with Dunnett's post test (GraphPad Prism 5.01, GraphPad Software, San Diego, CA, USA). Repeated measures ANOVA, with Dunnett's post test was used for the data in Fig. 2B. Curve fitting (Fig. 1D) was performed using the least-squared fitting algorithm implemented in SigmaPlot 10.0 (SYSTAT Software Inc., San Jose, CA, USA).

All chemicals were obtained from Sigma Aldrich (Gillingham, Dorset, UK) except: TTX (Latoxan; Valence, France) and bicuculline, NBQX, APV and MK801 (Tocris BioScience; Bristol, UK).

## Results

### Glutamine induces an inward membrane current in MNTB neurons

Principal neurons of the MNTB in rat brain stem slices were whole-cell patch-clamped at −70 mV and electrogenic glutamine transport currents were stimulated by the puff application of extracellular glutamine from either of two glass pipettes positioned close to the cell soma (Fig 1A). Pressure ejection of 10 mM l-glutamine on to the cell soma, produced an inward current (I_gln_) of −20.6±0.9 pA (*n*=61; example current shown in Fig. 1B). There was no significant run-down or run-up of the current magnitude following whole-cell recording, with stable recordings being observed for many 10s of minutes (Fig. 1C). The EC_50_ of I_gln_ was estimated by applying different glutamine concentrations to the neurons, using the two puffer pipettes with one containing 10 mM glutamine and the other containing 1, 5 or 20 mM. The dose–response curve is shown in Fig. 1D, fitted with a Michaelis–Menten equation (*K*_m_=0.94±0.25 mM).

### The glutamine current is mediated by an electrogenic transporter

If I_gln_ is mediated by a transporter, external glutamine application would be expected to produce an inward current at all membrane voltages. We therefore investigated the current–voltage (I/V) relationship of I_gln_ by varying the holding potential (from −80 mV to +40 mV in 20 mV steps) prior to, and for the duration of, the glutamine application. I_gln_ is reduced, but still inward, at positive membrane potentials, with an average magnitude of −11±2 pA at +40 mV (Fig. 2A; *n*=6, *P*<0.001), i.e. a 47±3% reduction of the current observed at −80 mV (Fig. 2B). A sample I/V curve from one cell is show in Fig. 2C. It is evident that I_gln_ does not reverse at depolarized potentials, which is consistent with an electrogenic transporter and rules out the involvement of a significant ion channel component to the current.

### The glutamine transport current is sodium-dependent

The majority of glutamine transport systems rely on the transmembrane sodium gradient to power the uptake process. Consequently, we investigated the sodium-dependence of I_gln_ by removing all of the external sodium and replacing it with choline. I_gln_ evoked by 10 mM glutamine application in normal (152 mM) sodium was −27.1±1.0 pA, and this was completely eliminated (−0.2±1.0 pA; *n*=7; *P*<0.001) by the removal of external sodium (Fig. 3). The total reliance of I_gln_ on external sodium clearly demonstrates the sodium-dependent nature of the glutamine transport process.

### Contamination of the glutamine by glutamate

MNTB somata express a high number of ionotropic glutamate receptors, and exhibit excitatory synaptic currents of approximately 10 nA in magnitude (Barnes-Davies and Forsythe, 1995; Borst et al., 1995; Schneggenburger et al., 1999). The relatively high affinity of the glutamate receptors (2–20 μM; Patneau and Mayer, 1990) means that even a very small contamination of the 10 mM glutamine solution with glutamate would cause a substantial glutamate-mediated current. To guard against this possibility we investigated the magnitude of the glutamate-induced current, and inhibited it pharmacologically. To simulate a 0.05% glutamate contamination, as has been previously observed with commercially sourced glutamine (Sands and Barish, 1989; Yamada and Rothman, 1989), 5 μM l-glutamate was applied to MNTB neurons by puff application (Fig. 4A). An inward current of −7.6±0.9 pA was observed (Fig. 4A_1_; *n*=5), which was completely inhibited (to −0.1±0.1 pA) by a cocktail of channel inhibitors containing 40 μM APV, 10 μM MK801, 10 μM bicuculline, 1 μM strychnine, 1 μM TTX and 20 μM NBQX (Fig 4A_2_ and 4 A_3_; *n*=5; *P*<0.001). However, in contrast, the glutamine induced current was not eliminated by the channel inhibitors (Fig. 4B). I_gln_ (−24±3 pA; Fig. 4B_1_) was slightly reduced (−20±2 pA) by the channel inhibitor cocktail (Fig. 4B_2_; *n*=8; *P*<0.01). This 4 pA (17%) reduction (Fig. 4C) is likely to be the result of a minor (<0.05%) glutamate contamination in the glutamine solution. All other electrophysiological experiments (Figs. 1–5) were performed in the presence of this cocktail of inhibitors. Under these conditions, I_gln_ has neither the electrical properties (Fig. 2), ion dependence (Fig. 3) nor pharmacological profile (Fig. 4) of a glutamate receptor-mediated current. A small amount of contaminating glutamate could possibly activate the electrogenic high-affinity excitatory amino acid transporters (EAATs). The transporter EAAT1 (also called glutamate/aspartate transporter-GLAST) is strongly expressed in the glia cells surrounding the MNTB principal cells (Renden et al., 2005). However, the EAAT3 (excitatory amino acid carrier 1-EAAC1) transporter, which is known to be present in the somatodendritic compartment of post synaptic neurons (Kanai and Hediger, 1992; Rothstein et al., 1994; Danbolt, 2001), is less strongly expressed in the MNTB (Renden et al., 2005). To explore the possible involvement of these transporters in the observed membrane current, we applied the broad-spectrum EAAT inhibitor dl-threo-b-benzyloxyaspartate (dl-TBOA; Shimamoto et al., 1998). Application of 200 μM dl-TBOA had no significant effect on I_gln_, which was −14.9±2.4 pA in control and −14.5±1.8 pA in the presence of TBOA (99±4% of control; Fig. 4D; *n*=4, *P*>0.05). The glutamine evoked current is therefore not a result of activation of EAATs, either by glutamine itself or by a small amount of contaminant glutamate.

### The glutamine currents are mediated by system A transport activity

To elucidate which glutamine transport system is responsible for generating the currents in MNTB neurons, we investigated the pharmacological properties of I_gln_. The two main electrogenic, sodium-dependent, glutamine transporters expressed in neurons are system A and system B^0^. System A transporters are characteristically sensitive to the competitively transported amino acid analogue *N*-(methylamino)isobutyric acid (MeAIB; Christensen et al., 1965), and are unaffected by 2-aminobicyclo-[2.2.1]-heptane-2-carboxylic acid (BCH; Christensen, 1990). In contrast, system B^0^ transporters are competitively inhibited by BCH, but are unaffected by MeAIB (Sloan and Mager, 1999; Broer et al., 2004, 2006). We therefore examined MeAIB-induced currents and I_gln_ in the presence of MeAIB or BCH (Fig. 5). Puff application of 10 mM MeAIB induced an inward current of −7.4±0.8 pA (Fig. 5A_2_), which is 34±2% of I_gln_ (Fig. 5B; *n*=4; *P*<0.001), consistent with MeAIB being a transported substrate for this carrier with a slower maximal transport rate. Additionally, glutamine and MeAIB can be shown to be acting on the same transporter as the continued presence of MeAIB in the bathing solution occludes I_gln_: 10 mM MeAIB reduced I_gln_ to −3.6±0.4 pA (by 80±3%; Fig. 5A_*3*_; *n*=15, *P*<0.001), and 20 mM reduced it to −1.0±0.4 pA (by 96±2%; Fig. 5A_*4*_; *n*=4, *P*<0.001). Ten millimolar BCH had no significant effect on the current, with I_gln_ being −15.6±2.1 pA (84±4% of control; Fig. 5A_*5*_ and *B*; *n*=6, *P*>0.05). These results indicate that I_gln_ is mediated by system A transport and not by system B^0^.

Two isoforms of the system A transporters have been proposed to be present in neurons, SAT1 and SAT2. While they both transport glutamine, they differ in their transport rates for l-proline. The proline transport rate of SAT2 is greater than glutamine transport rate, showing an increase in current of between 10 and 100% (Reimer et al., 2000; Chaudhry et al., 2002b; Mackenzie et al., 2003). The proline transport rate for SAT1 is between 60 and 90% lower than the glutamine transport rate (Chaudhry et al., 2002b; Mackenzie et al., 2003). By alternately applying 10 mM of either glutamine or l-proline via the two puffer pipettes, we investigated the relative magnitude of the proline current. The proline-induced current (I_pro_) was a similar magnitude to I_gln_ (−24.8±2.6 pA; 116±7% of control; Fig. 5A_*6*_ and *B*; *n*=12, *P*>0.05). MeAIB (10 mM) reduced I_pro_ to −5.8±1.7 pA (by 72±6%; Fig. 5A_*6*_ and *B*; *n*=4, *P*<0.001), which is comparable to the competitive inhibition of I_gln_. The relatively large magnitude of I_pro_ and its inhibition by MeAIB indicate that at least part of I_gln_ must be mediated by SAT2, but does not rule out the possibility that both SAT1 and SAT2 contribute to the glutamine current in these cells. In contrast to l-proline, d-proline induces a greatly reduced current (−2.1±0.9 pA; Fig. 5B; *n*=5; *P*<0.001), consistent with the stereo-specificity of system A transport (Varoqui et al., 2000).

### SAT1 and SAT2 proteins are expressed in the MNTB

**Fig. 6:**
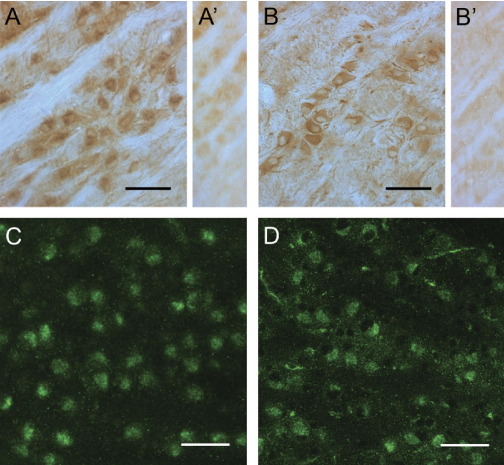
Two system A transporters, SAT1 and SAT2, are localized to the cell bodies of the principal neurons of the MNTB. Coronal sections were cut through the rat auditory brainstem and immunoperoxidase-stained using affinity-purified antibodies generated against SAT1 and SAT2. (A): Strong SAT1 immunoreactivity is detected in neuron-like cell bodies in the adult MNTB leaving the trapezoid body fibers unstained. (A′) This staining pattern is abolished upon preincubation of the antibodies with the immunizing GST fusion protein. (B) SAT2 immunoreactivity is also pronounced in the cell bodies of scattered neurons in between trapezoid body fibers in the adult MNTB. (B′) Only faint diffuse background staining remained in this nucleus upon preincubation of the SAT2 antibody with the GST fusion protein used to immunize the rabbits. C and D: Fluorescent immunocytochemical staining of SAT1 (C) and SAT2 (D) in 13–14 day old rats, showing strong labeling in MNTB principal cells bodies. The scale bar in all panels is 50 μm.

To further investigate which of the system A transporter isoforms contribute to I_gln_ in the MNTB, we performed immunocytochemical staining using antibodies raised against SAT1 and SAT2 proteins. Immunoreactivity for both SAT1 (Fig. 6A, C) and SAT2 (Fig. 6B, D) was detected in large neuronal-like cell bodies in the MNTB, indicating the presence of these transporters in the principal neurons. No reactivity was observed in the axons of the trapezoid body fibers, which traverse this brain region. The staining is abolished by preincubation of the antibodies with the GST fusion protein used during the immunization of the rabbits (Figs. 6A′, B′), which confirms the specificity of the antibodies for SAT1 and SAT2. Combined with the electrophysiological data, this provides strong evidence that both SAT1 and SAT2 contribute to the glutamine-induced current that we observe in these neurons.

## Discussion

We have shown that MNTB neurons use membrane transporters to take up glutamine in a sodium-dependent and electrogenic manner. The current-voltage relationship reveals that the current induced by external glutamine is always inward, even at considerably positive membrane voltages. This is indicative of a transporter current, and is consistent with the inward rectification observed for system A transporters expressed in *Xenopus* oocytes or HEK293T/17 cells (Chaudhry et al., 2002b; Mackenzie et al., 2003; Zhang and Grewer, 2007). The observed I_gln_ is not an artifact of glutamate contamination of the glutamine solution, as it does not have the pharmacology of a glutamate transporter or receptor mediated current, and the voltage dependence is inconsistent with activation of a non-specific cation channel.

The electrical and pharmacological profile of the current is consistent with it being mediated by system A transport, and excludes the involvement of all other transport systems: the glutamine current is sodium-dependent, unlike the transport mediated by systems L (Kanai et al., 1998; Rossier et al., 1999; Segawa et al., 1999) and b^0,+^ (Bertran et al., 1992; Tate et al., 1992; Wells and Hediger, 1992). Of the sodium-dependent transporters, ASCT2 (Utsunomiya-Tate et al., 1996; Broer et al., 1999) and y^+^L (Broer et al., 2000) are electroneutral, so cannot contribute to the observed current. The system N transporters SN1 and SN2 are sodium-dependent and can be electrogenic as a result of an uncoupled proton flux (Chaudhry et al., 1999, 2001; Broer et al., 2002). Although they are highly expressed in the MNTB, expression is limited to glial cells and they are absent from the MNTB principal neurons (Boulland et al., 2002; Cubelos et al., 2005). All of these transporters (L, b^0,+^, ASCT2, y^+^L and N) do not transport proline and are insensitive to MeAIB. The system B^0^ transporter B^0^AT2 does transport glutamine and proline in an electrogenic, sodium-dependent manner. However, it cannot be the mediator of the current we observe as B^0^AT2 is insensitive to MeAIB and is inhibited by BCH. In addition, the transport efficiency for proline is much higher than that for glutamine (Takanaga et al., 2005; Broer et al., 2006), which is contrary to our observations. The related system B^0,+^ transporter ATB^0,+^ is also an electrogenic glutamine transporter. However, its expression in the brain is limited, it is sensitive to BCH and it does not transport proline (Sloan and Mager, 1999). System A is therefore the only transport system that fulfills the criteria of sodium-dependent, electrogenic transport of glutamine and proline, in an MeAIB sensitive and BCH insensitive manner. The MeAIB induced current is 34% of the magnitude of I_gln_, which is very similar to the relative MeAIB current observed in *Xenopus* oocytes expressing SAT1 (Chaudhry et al., 2002b) or SAT2 (Reimer et al., 2000; Sugawara et al., 2000a; Yao et al., 2000). In addition to system A, the proton/amino acid transporters PAT1 and PAT2 have also been shown to transport MeAIB (Chen et al., 2003ab; Kennedy et al., 2005), however unlike the currents that we observe, they are sodium-independent and not selective for l-over d-proline (Sagne et al., 2001; Boll et al., 2002; Wreden et al., 2003; Foltz et al., 2004). These results additionally implicate system A as the sole mediator of I_gln_.

Which isoforms of the system A family could be involved in producing the membrane current we observe? System A transporters belong to the solute carrier *SLC38* gene family (Sundberg et al., 2008), three members of which (*SLC38A1*, *A2* and *A4*) have been ascribed to system A. The *SLC38A1* transporter protein (SAT1) is expressed mainly in neurons (Varoqui et al., 2000; Wang et al., 2000; Chaudhry et al., 2002b; Mackenzie et al., 2003; Melone et al., 2004; Buntup et al., 2008) whereas the *SLC38A2* protein (SAT2) is expressed in many tissues, including neurons and potentially in glia (Reimer et al., 2000; Sugawara et al., 2000a; Yao et al., 2000; Gonzalez-Gonzalez et al., 2005; Melone et al., 2006; Jenstad et al., 2009). In contrast, the *SLC38A4* transporter (SAT3, SNAT4 or ATA3) is exclusively located in liver, skeletal muscle, placenta and olfactory bulb (Sugawara et al., 2000b; Hatanaka et al., 2001a; Desforges et al., 2006), and not in the brainstem (Sundberg et al., 2008). Our immunocytological data indicate the presence of two glutamine transporter isoforms, SAT1 and SAT2, in MNTB neurons. This finding is supported by our electrophysiological data, which show a proline response that is neither significantly greater (as expected for SAT2) nor significantly smaller (as for SAT1) than the glutamine response. In addition, we observe a *K*_m_ for glutamine of 0.94±0.25 mM, which lies in between the *K*_m_s observed for SAT1 (0.3–0.4 mM) and SAT2 (1.7–2.3 mM) when expressed in *Xenopus* oocytes (Yao et al., 2000; Chaudhry et al., 2002b; Mackenzie et al., 2003). The magnitude of the proline current and intermediate glutamine affinity are consistent with a mixture of SAT1 and SAT2 currents in our recordings. While membrane currents mediated by system A transporters have previously been shown in single cell expression systems, our study is the first to demonstrate system A transporter currents recorded in neurons *in situ*.

What is the role of glutamine transport into neurons? Under normal physiological conditions it is thought that glutamine is transported out of glial cells by system N transport and into neurons by system A (Chaudhry et al., 1999, 2002a; Jenstad et al., 2009). A similar system N—system A glutamine shuttle has also been proposed to occur in the pancreas, where it may be involved in the regulation of insulin secretion (Gammelsaeter et al., 2009). Our current finding of SAT1 and SAT2 localization in the principal neurons of MNTB surrounded by SN1 and SN2 expressing glial processes (Boulland et al., 2002; Cubelos et al., 2005) corroborate the complementary expression of the system N and system A transporters seen in other brain regions and consent with the notion that these transporters work in concert to transport glutamine from glial cells to neurons (Chaudhry et al., 2002a). Moreover, inhibition of system A *in vivo* results in a 1.8 fold rise in the extracellular glutamine concentration and dramatically reduces the intracellular concentrations of glutamate (Kanamori and Ross, 2004; Jenstad et al., 2009), underlining the importance of system A transport in amino acid flux into neurons (Rae et al., 2003). Since neurons lack pyruvate carboxylase (Yu et al., 1983; Shank et al., 1985), they are thought incapable of anaplerotic replenishment of TCA cycle intermediates from glucose. It is therefore vital that they are able to import longer carbon chain molecules, such as glutamine, to replenish those lost through release of neurotransmitters or through other metabolic processes. In support of this notion, it has been observed that approximately 50% of the glutamine applied to cultured neurons is metabolized via the TCA cycle (Waagepetersen et al., 2005). Glutamine has been proposed to be directly utilized as a precursor for the neurotransmitter glutamate, in the classically described glutamate–glutamine cycle (Benjamin and Quastel, 1972; Hamberger et al., 1979). This has been shown to occur under physiological conditions in the retina (Barnett et al., 2000; Poitry et al., 2000) and in hippocampal cell culture and slices (Armano et al., 2002; Bacci et al., 2002), although this hypothesis has recently been challenged (Kam and Nicoll, 2007). Glutamine, via the production of glutamate, is also a precursor for the inhibitory transmitter GABA (Sonnewald et al., 1993), and system A transport in this pathway has been demonstrated to contribute to the maintenance of inhibitory synaptic transmission in the hippocampus (Liang et al., 2006; Fricke et al., 2007). The MNTB neurons used in this study are principally glycinergic, but also release GABA as a co-transmitter (Webster et al., 1990; Chaudhry et al., 1998; Kotak et al., 1998; Korada and Schwartz, 1999; Smith et al., 2000). Additionally, they have been shown to release glutamate in a vesicular manner from their presynaptic terminals, which is most prominent early on in development (post-natal days 1–8), but also persists at older ages (Gillespie et al., 2005). It is therefore likely that the transport of glutamine into these neurons is involved in maintenance of their neurotransmitter pool. This glutamate release is mediated by the vesicular glutamate transporter 3 (VGLUT3), which is expressed both in the presynaptic terminals and the MNTB somata (Boulland et al., 2004; Gillespie et al., 2005). Somatodendritic expression of VGLUT3 could also mediate glutamate release as a retrograde messenger (Harkany et al., 2004), with a mechanism that has previously been shown to be dependent on system A glutamine transport into neurons (Jenstad et al., 2009).

As a consequence of its important role in amino acid flux and cell metabolism, system A transport is noted for its high degree of regulation by a number of environmental stimuli (McGivan and Pastor-Anglada, 1994). These include downregulation in ischemic preconditioning (Kamphuis et al., 2007), upregulation in response to starvation or amino acid deprivation (Kilberg et al., 2005), subtype dependent regulation by agents that increase cAMP (Hatanaka et al., 2001b), and a rapid induction of expression of SAT1 by brain-derived neurotrophic factor (BDNF; Burkhalter et al., 2007). Glutamine that is transported into cells (by system A; Baird et al., 2009) is also used to power tertiary active transport of essential amino acids (such as leucine) via system L, which then go on to activate signaling pathways such as the mammalian target of rapamycin (mTOR; Baird et al., 2009; Nicklin et al., 2009). Its involvement in these signaling pathways places system A at the heart of many cellular physiological processes. Hence, our demonstration of neuronal system A transporter currents *in situ* will allow further investigation of the role that this transporter plays in coordinating the cellular response to neuronal activation.
